# Evaluation of Amino Acid Profiles of Rice Genotypes under Different Salt Stress Conditions

**DOI:** 10.3390/plants12061315

**Published:** 2023-03-14

**Authors:** Muhammad Farooq, Yoon-Hee Jang, Eun-Gyeong Kim, Jae-Ryoung Park, Gyu-Hyeon Eom, Dan-Dan Zhao, Kyung-Min Kim

**Affiliations:** 1 Department of Applied Biosciences, Kyungpook National University, Deagu 641566, Republic of Korea; 2 Crop Breeding Division, National Institute of Crop Science, Rural Development Administration, Wanju-gun 55365, Republic of Korea; 3 Crop Foundation Research Division, National Institute of Crop Science, Rural Development Administration, Wanju-gun 55365, Republic of Korea

**Keywords:** essential amino acids, non-essential amino acids, origin, genetic base, immune level

## Abstract

Amino acids are building blocks of proteins that are essential components of a wide range of metabolic pathways in plant species, including rice species. Previous studies only considered changes in the amino acid content of rice under NaCl stress. Here, we evaluated profiles of essential and non-essential amino acids in four rice genotype seedlings in the presence of three types of salts, namely NaCl, CaCl_2_, and MgCl_2_. Amino acid profiles in 14-day-old rice seedlings were determined. The total essential and non-essential amino acid contents in cultivar Cheongcheong were considerably increased upon NaCl and MgCl_2_ application, whereas total amino acids were increased upon NaCl, CaCl_2_, and MgCl_2_ application in the cultivar Nagdong. The total amino acid content was significantly lower in the salt-sensitive cultivar IR28 and salt-tolerant Pokkali under different salt stress conditions. Glycine was not detected in any of the rice genotypes. We observed that cultivars with the same origin respond similarly to each other under salinity stress conditions: cultivars Cheongcheong and Nagdong were found to show increased total amino acid content, whereas the content in foreign cultivars IR28 and Pokkali was found to decrease. Thus, our findings showed that the amino acid profile of each rice cultivar might depend on the origin, immune level, and genetic makeup of the respective cultivar.

## 1. Introduction

Rice is the primary source of food for more than half of the world’s population. However, rice is a glycophyte, i.e., its growth and development are negatively affected by environmental stress factors such as high soil salinity. Rice plants are thus highly prone to salinity stress, particularly in reproductive and seedling stages [1,2]. Around 1 × 10^9^ hectares of land is affected by adverse salinity conditions worldwide, which accounts for about 20% of total agricultural land. Moreover, the area affected by salinization continues to expand. Nearly half of all agricultural land around the world is estimated to be salinized by 2050 [3,4]. Different salts such as NaCl, Na_2_SO_4_, MgCl_2_, and CaCl_2_ deleteriously affect the germination percentage and rate of germination of *Pinus halepensis* Mill. (Aleppo pine). However, a salt with a low MgCl_2_ concentration exhibits the highest gemmation percentage when compared to other salts [5]. It was reported that the cool-season grasses, tall fescue (*Festuca arundinacea* Schreb.), show significant tolerance to CaCl_2_, NaCl, and MgCl_2_ salts, whereas warm-season grasses, bermudagrass (*Cynodon dactylon* (L.) Pers.), show maximum tolerance to CaCl_2_ and NaCl stress only [6]. NaCl stress may prevent protein synthesis, which disturbs amino acid metabolisms and the nutritional value of the plant product [7].

Under salinity stress, sodium (Na^+^) and Chloride (Cl^−^) enter the root system from the surrounding saline environment and are transported, via the xylem vessels of the transpiration stream, to the shoots. Because only a little amount of Na^+^ can be returned to the roots via the phloem, these ions build up and accumulate in the shoots [8,9]. For most species, including cereals, Na^+^ accumulation in the shoots results in ion toxicity [9]; however, for some species such as citrus, grapevine, and soybean it is Cl^−^ which accumulates up to hazardous levels in the shoots before Na^+^ [10,11]. High levels of Na^+^ ions in the shoots can disrupt cellular and metabolic processes that occur within the cells of the shoots. Sodium (Na^+^) ions and potassium ions (K^+^) have similar physiochemical properties; therefore, Na^+^ is proposed to interfere with a wide range of processes regulated by K^+^ [12].

Salt stress increased valine accumulation while decreasing tyrosine accumulation in *Lygeum spartum* L. (Poaceae) [13]. Osmotic stress and ion toxicity are major consequences of salt stress. This effect subsequently leads to oxidative stress. Plants respond to salt stress via various mechanisms, including by selective absorption of K^+^ over Na^+^ ions, and the export of reactive oxygen species (ROS) via the antioxidant defense system [14,15,16]. Additionally, the small organic solutes such as glucose, glycerol, glycine, inositol, polyamines, sugars, and betaine are produced to increase the osmotic pressure of the cell to counter salinity stress [17,18]. Salt stress has also been found to increase the content of proline in the root and nodules of the alfalfa plant (*Medicago sativa*) [19].

Amino acids are vital ingredients in human nutrition. However, the levels of several amino acids in grains of widely-consumed cereals such as rice and wheat are low. So-called “essential amino acids,” such as plant-based lysine (Lys), isoleucine (Ile), leucine (Leu), Met, valine (Val), phenylalanine (Phe), tryptophan, and cysteine, contribute greatly to nutritional diets. Furthermore, some free amino acids may alter fruit flavor. The most well known is _L_-Glu, which is responsible for the ‘umami’ or delightful flavor [20]. Alanine (Ala) or Lys are associated with sweetness, whereas Phe or tyrosine (Tyr) are correlated with bitterness [21]. Under salt stress, proline content in rice varieties of Aychade, Fidji, and Giano was found to increase by 10 to 50 times compared to 2AP (2-acetyl-1-pyrroline) [22]. Salt stress disrupts the metabolic balance in rice, hence the proline contents of rice varieties were found to increase significantly in all rice genotypes in the presence of 120 mM of salt [23]. Previous studies also reported that both drought conditions and high salt content in the soil lead to increased proline content in many plants compared to levels of other amino acids. Several studies further suggested that increased proline content helps protect the cell membrane against salt injury, whereas proline accumulation under salt stress was proposed to show a negligible effect on osmotic adjustment as well [23,24].

Soil salinity is one of the major yield-limited factors, is derived from underground rock salt, and is predicted to expand in the future. Since so many nations, including those in North America, Africa, Asia, the Pacific, Europe, Latin America, and Thailand, are addressing this issue, controlling the impact of salinity on crops is crucial and, therefore, controlling the impact of salinity on crops requires special attention. Traditionally, amino acids have been considered the building blocks of protein and an essential nutrient for all living organisms. Soil salinity significantly altered the amino acid profile, as a result causing modification in protein structure. Therefore, in the present study, we evaluate the amino acids’ profile, which can be classified into essential, non-essential, and conditional amino acids. Among the 20 naturally occurring amino acids, 9, 11, and 6 are essential, non-essential, and conditional amino acids that are required under stress conditions, respectively. Most of the previous studies focused only on changes in profiles of either one or two amino acids under NaCl stress in rice. Here, we analyzed profiles of 16 amino acids in four different rice genotypes under stress induced by the presence of three types of salts (NaCl, CaCl_2_, and MgCl_2_) to investigate the effect of salt type on the amino acid content of rice cultivars.

## 2. Results

### 2.1. Effect of Different Salt Types on Essential Amino Acid Profiles

Seven essential and eight non-essential amino acids were detected, which corresponds to a total of 15 amino acids out of the possible 20. We identified histidine, isoleucine, leucine, lysine, methionine, phenylalanine, and valine as essential amino acids under different salt stress conditions. Both NaCl and MgCl_2_ applications were found to significantly increase the amounts of essential amino acids histidine, isoleucine, lysine, methionine, phenylalanine, and valine in cultivar Cheongcheong compared to other cultivars (Table 1). Similarly, CaCl_2_ stress dramatically increased histidine, isoleucine, lysine, methionine, phenylalanine, and valine levels in the cultivar Nagdong (Table 1). However, when compared to Cheongcheong and Nagdong cultivars, the salt-sensitive cultivar IR28 and salt-tolerant cultivar Pokkali had considerably lower amounts of all essential amino acids under different salt stress conditions (Table 1). The CaCl_2_ salt significantly reduced all essential amino acids in cultivar IR28, whereas MgCl_2_ salt decreased all essential amino acids in cultivar Pokkali (Table 1).

### 2.2. Effect of Different Salts on Non-Essential Amino Acids

Levels of non-essential amino acids were found to increase or decrease depending on rice genotypes under different salt stress conditions. However, levels of non-essential amino acids alanine, aspartic acid, cysteine, glutamic acid, proline, and tyrosine were found to increase significantly in cultivar Cheongcheong under MgCl_2_ stress (Table 2). On the other hand, NaCl stress was also found to induce cultivar Cheongcheong to produce more alanine, cysteine, proline, serine, and tyrosine compared to their respective control groups. Different types of salts were found to lead to higher levels of alanine, cysteine, glutamic acid, proline, serine, and tyrosine in the cultivar Nagdong (Table 2). Salt-sensitive cultivar IR28 showed significant decreases in levels of alanine, aspartic acid, arginine, glutamic acid, and proline under different salt stress (Table 2). Serine level, on the other hand, was found to increase significantly under MgCl_2_ stress. Similarly, the alanine and aspartic acid levels were found to increase in cultivar Pokkali upon the application of different salt types, whereas levels of other amino acid types were found to show significant decreases (Table 2).

### 2.3. Effect of Different Salt Types on Total Essential and Non-Essential Amino Acid Profiles

**Figure 1:**
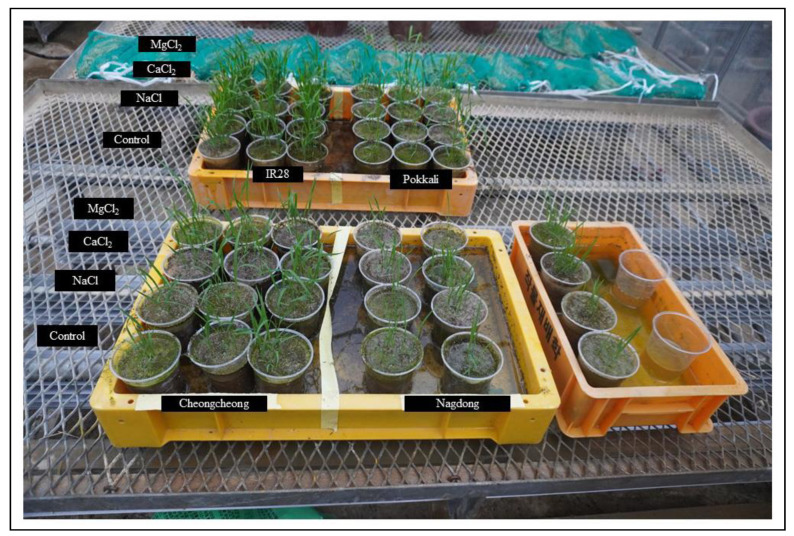
Effect of salinity stress conditions on total amino acid profiles in rice genotypes. Total essential and non-essential amino acid contents were determined after 24 h of 150 mM salinity stress. (**A**,**B**,**E**,**F**) are cultivars Cheongcheong and Nagdong, whereas (**C**,**D**,**G**,**H**) show cultivars IR28 and Pokkali. Different characters on the bars indicate significant differences. However, Duncan’s multiple range test, *p* 0.05, shows that the means of the similar letters are not statistically different.

Different salt types affected the profiles of total essential and non-essential amino acids differently in rice genotypes. The NaCl and MgCl_2_ applications were found to significantly increase both essential and non-essential amino acids in cultivar Cheongcheong (Figure 1A,E). However, in cultivar Nagdong, NaCl, CaCl_2_, and MgCl_2_ salts were found to increase levels of both essential and non-essential amino acids significantly (Figure 1B,F). On the other hand, salt-sensitive cultivar IR28 and salt-tolerant cultivar Pokkali were found to show significantly decreased total essential and non-essential amino acid levels under different salt stress (Figure 1C,D,G,H). It was observed that cultivars of the same origin respond almost identically to different salt stresses. Similarly, cultivars IR28 and Pokkali reduced the total essential and non-essential amino acids, but cultivars Cheongcheong and Nagdong increased the total essential and non-essential amino acids significantly (Figure 1).

## 3. Materials and Methods

### 3.1. Plant Materials and Growth Conditions

**Figure 2:**
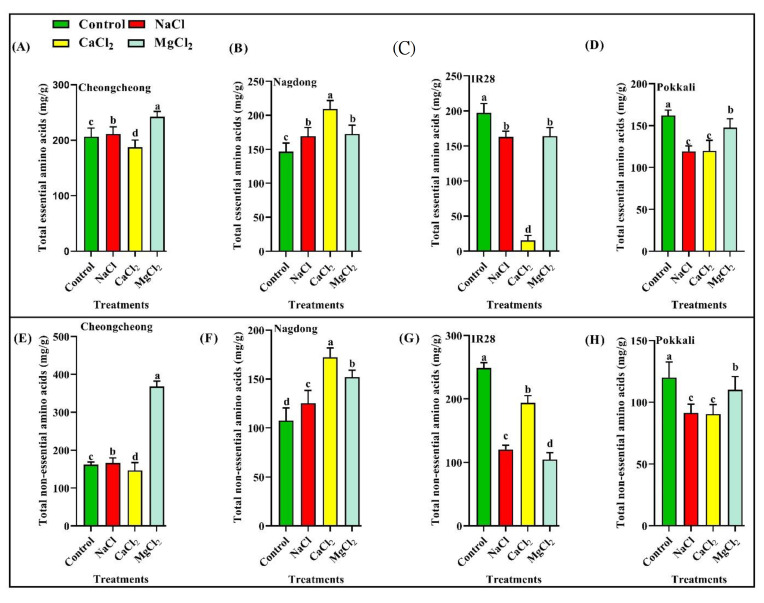
Rice seedlings growing in a greenhouse. After 14 days, treatment with 150 mM of different salt types was applied for 24 h.

Four rice genotypes, namely Cheongcheong, Nagdong, IR28, and Pokkali, were selected for the study. Cultivars Cheongcheong and IR28 were previously found [25] to be salt-sensitive, whereas cultivars Nagdong and Pokkali were found to be salt-tolerant. Seeds of all rice genotypes were first surface sterilized with 70% ethanol. Then, the seeds were placed in a 5% sodium hypochlorite (NaOCl) solution and kept for 15 min. The sterilized seeds were then dried using clean tissue paper. After that, the seeds of each cultivar were allowed to pre-germinate in Petri dishes for three to four days. When the seeds of all rice cultivars germinated successfully, 10 germinated seeds were transferred to 500 mL closed plastic water cups containing soil. The soil used here is N-P rich, with concentrations of 800–2500 mg/kg nitrogen and 150–650 mg/L phosphorus. The germinated seeds were grown for two weeks in the greenhouse under 30 °C for 16 and 8 h of daily light and dark periods, respectively. Three replicates for each treatment were made and kept in a yellow tray containing tap water to maintain the plastic cups’ temperature in the greenhouse during the summer season (Figure 2). In order to prevent the mixing of the salt solution with the external media, we used plastic pots with closed bottoms.

### 3.2. Preparation and Application of Salt Solutions

The rice seedlings develop two to three leaves within two weeks. After that, three salt solutions, each including 150 mM NaCl, CaCl_2_, or MgCl_2_, and water with no salt were used as a control. To make a 150 mM salt solution we dissolved separately 4.383 g NaCl, 8.3235 g CaCl_2_, and 7.1408 g MgCl_2_ in 500 mL distilled water using graphpad.com/quickcalscs/molarityform/. Finally, rice cultivars were treated with 300 mL of each salt solution with 150 mM concentration for 24 h. After harvesting the rice seedlings, they were kept immediately at −80 °C in a refrigerator. Afterward, the samples were freeze-dried in an incubator for seven days, and the amino acid contents were determined.

### 3.3. Analysis of Amino Acid Profiles

We used a method previously described by Farooq et al. (Farooq et al., 2020) to analyze the amino acid profiles of rice seedlings under different salt stress conditions. We used a total of thirty seedlings in three replicates and afterward selected fifteen seedlings. Briefly, samples of rice seedlings were ground (100 mg) and hydrolyzed first in 6 N HCl under vacuum at 110 °C for 24 h, then at 80 °C for 24 h. Before being injected into the amino acid analyzer (Ezchrom Elite for Hitachi L-8900, Tokyo, Japan), dried residues were homogenized in 0.02 N HCl, and passed through a 0.45 m filter. Ammonia (gas) is produced during the analysis procedure. Therefore, ammonia was excluded from the analysis.

### 3.4. Statistical Analysis

Three replicates were performed for each group. The mean and standard deviation of the data were determined using SPSS software (IBM SPSS Statistics, version 22, Redmond, WC, USA). Then, statistically significant differences were determined by performing one-way ANOVA and Duncan’s multiple range test (DMRT).

## 4. Discussion

Here, we investigated the effects of three different salt types on the amino acid profiles of four different rice genotypes. L-histidine, a common amino acid found in proteins, is essential for the growth and development of plants. The regulation of plant development in response to hormones and environmental factors is greatly influenced by histidine kinases (HK) [26]. A recent study describes that histidine reduces the level of reactive oxygen species (ROS) and improves the plants’ tolerance towards salt stress [27]. In our study, we find that MgCl_2_ and CaCl_2_ salts increase the histidine content in cultivars Cheongcheong and Nagdong, which might lead to a decrease in ROS levels in these cultivars.

A previous study also found that when exposed to NaCl stress and clinostat rotation, levels of all amino acids increase significantly [28]. We also found that the application of three salt types (NaCl, CaCl_2_, and MgCl_2_) lead to significantly increased amino acid levels in cultivars Cheongcheong, and Nagdong. It has been reported that 6% salinity (1098.79 μM g^−1^ DW) leads to increased levels of total amino acids in the root exudates of *Phragmites australis* [29]. Previous investigation suggests that the essential amino acids include alanine, arginine, glycine, leucine, serine, and valine and the non-protein amino acids are citrulline and ornithine [30,31]. It was reported that 10% NaCl stress significantly increases the free amino acids content in rhizome of *Phragmites australis* (Cav.) Trin. ex Steud [32]. It was reported that the quinoa genotype (Q5) shows unchanged essential amino acids content under salinity stress, and there is a positive correlation of Na^+^ with Pro, Gly, and Isoleucine [33]. Many studies revealed that when plants are exposed to high salinity, they respond to the resulting osmotic stress by increasing levels of proline significantly [34]. However, in the current study we did not detect glycine in rice genotypes, whereas the contents of proline and isoleucine were regulated differentially by various salt among rice genotypes. A previous study reported that under salinity stress Strawberry cvs Elsanta and Korona greatly increase the total amino acids, especially essential amino acids [35]. Here, we determined a significant increase in levels of total essential and non-essential amino acids under NaCl and MgCl_2_ stresses. However, under CaCl_2_ stress, a large increase in amino acid levels was observed only for the cultivar Nagdong (Table 1). A recent study reported that 1.0% to 2.0% NaCl stress significantly decreases the level of aspartate, alanine, histidine, glycine, isoleucine, leucine, lysine, phenylalanine, serine, and tyrosine in *Pleurotus Ostreatus* fruiting bodies [36]. In the present study, we also found that NaCl stress decreases both essential and non-essential amino acids in all rice genotypes except cultivar Cheongcheong.

Evidence suggests that endogenous cysteine (Cys) levels have risen when plants are subjected to environmental stresses such as chemical toxicity, heavy metals, and salt stress [37]. Here, we also found the high content of cystine in cultivar Cheongcheong and IR28 under MgCl_2_ and CaCl_2_ stress. However, both essential and non-essential amino acid levels decrease significantly in the salt-sensitive cultivar IR28 and salt-tolerant cultivar Pokkali under all salt stress conditions. It has been reported that salt stress caused increases in aspartic acid, arginine, glutamic acid, ornithine, proline, and γ-amino-butyric acid level [38]. Several studies reported that amino acids act as precursors for the synthesis of secondary metabolites and signaling molecules such as polyamines are derived from Arg [39], the plant hormone ethylene is synthesized from Met [40], and immune signaling requires conversion of Lys to N-hydroxy pipecoline [41,42]. In the present study, under different salt stress we found differential levels of arginine, aspartic acid, glutamic acid, and proline in rice genotypes. Some amino acids such as Lys and the branched-chain amino acids Val, Leu, and Ile have been identified as vital components of Arabidopsis’s dehydration tolerance [43]. Similarly, in the present study NaCl stress significantly up-regulates the Lys, Val, Leu, and Ile in cultivar Nagdong compared to other cultivars, and as well from their respective control group, suggesting that cultivar Nagdong have greater dehydration tolerance than other rice genotypes.

Proline is a cyclic amino acid with low molecular weight and is considered a major osmoprotectant, known to help maintain salinity tolerance in plants, protect membrane structure, and sustain enzyme/proteins activity [44,45]. The population of the salt-tolerant oilseed plant *Eruca sativa* Mill has considerably more proline, free amino acids, and soluble sugar than the non-tolerant population [46]. Our findings indicate that cultivars Cheongcheong and Nagdong display increased proline content under NaCl, MgCl_2_, and CaCl_2_ stress conditions. On the other hand, cultivars IR28 and Pokkali displayed decreased proline levels (Table 1). Based on our previous findings on both cultivars, we determined that Cheongcheong is a salt-sensitive cultivar, whereas Nagdong is a salt-tolerant cultivar [47]. On the other hand, IR28 and Pokkali are cultivars that are well known for their sensitivity and tolerance to salt stress, respectively. Although our findings suggest that proline may not play a substantial role in salinity tolerance, we identified a higher proline content in cultivars Cheongcheong and Nagdong under NaCl stress, yet lower proline levels in IR28 and Pokkali. Many studies suggest that under salt stress, increased exogenous proline levels increase the resistance of celery (*Apium graveolens L. cv.*SB 12) somatic embryos to partial dehydration [48]. Exogenous proline was previously found to boost the proliferation of salt-stressed tobacco cell cultures [49]. Hence, changes in amino acid levels under different salt stress conditions may be attributed to differences in metabolic pathways involved in different rice genotypes.

## 5. Conclusions

The cultivars Cheongcheong, Nagdong, and IR28 were found to show significantly increased levels of both essential and non-essential amino acids when exposed to MgCl_2_ and CaCl_2_ at 150 mM concentration. However, CaCl_2_ and MgCl_2_ applications were found to increase the levels of total essential and non-essential amino acids in cultivars Nagdong and Cheongcheong, respectively. Levels of both essential and non-essential amino acids were found to decrease significantly in cultivars IR28 and Pokkali. In general, these findings may be attributed to differences in the genetic background and immunity profiles of different rice genotypes. We found that cultivars of the same origin respond to salt stress similarly. For example, cultivars Cheongcheong and Nagdong exhibit increased amino acid content, whereas cultivars IR28 and Pokkali exhibit decreased levels.

## Figures and Tables

**Table 1:** Rice seedlings displayed differential regulation of essential amino acids following 24 h different salts stress. Different letters indicate significant differences between rice genotypes. Means ± SDs, *n* = 3; values with the same letter were not found to be significantly different from each other as per Duncan’s multiple range test at *p* < 0.05.

		Amino Acids Concentration (mg/g)							
	Amino Acids	Control		NaCl		CaCl_2_		MgCl_2_	
	histidine	21.9320	±0.0481 ^a^	22.3443	±0.0572 ^a^	18.6910	±0.0530 ^b^	26.2183	±0.0780 ^b^
	isoleucine	21.5630	±0.0278 ^a^	23.0637	±0.0405 ^a^	20.8033	±0.13930 ^b^	20.8923	±0.0810 ^a^
**Cheongcheong**	leucine	47.5327	±0.0476 ^d^	49.0367	±0.0449 ^d^	42.1950	±0.0040 ^b^	56.7487	±0.0366 ^d^
	lysine	47.5327	±0.0476 ^a^	49.0367	±0.0449 ^a^	42.1950	±0.0040 ^b^	56.7487	±0.0366 ^a^
	methionine	11.4197	±0.0830 ^a^	13.0113	±0.0080 ^a^	13.6030	±0.0538 ^b^	10.6220	±0.0726 ^a^
	phenylalanine	50.9810	±0.0135 ^a^	49.5547	±0.06824 ^a^	46.3273	±0.0479 ^b^	62.3823	±0.0910 ^a^
	valine	31.4523	±0.0605 ^a^	32.3437	±0.4789 ^a^	27.0763	±0.05525 ^b^	38.2525	±0.2092 ^a^
	histidine	12.4530	±0.0416 ^d^	13.0277	±0.0670 ^b^	20.2657	±0.0606 ^a^	18.4313	±0.0504 ^b^
	isoleucine	42.1503	±0.0513 ^d^	47.3487	±0.1185 ^c^	11.6133	±0.0671 ^a^	23.1907	±0.0082 ^b^
**Nagdong**	leucine	28.3263	±0.0971 ^c^	30.7597	±0.0577 ^a^	48.7727	±0.0193 ^c^	40.1517	±0.0504 ^b^
	lysine	28.3263	±0.097 ^d^	30.7597	±0.0577 ^c^	48.7727	±0.0193 ^a^	40.1517	±0.0504 ^b^
	methionine	6.1750	±0.01840 ^b^	8.8327	±0.0499 ^b^	14.3833	±0.4374 ^a^	5.5293	±0.0510 ^c^
	phenylalanine	27.4770	±0.0558 ^d^	36.6877	±0.0279 ^b^	62.4527	±0.0435 ^a^	40.8457	±0.0409 ^b^
	valine	17.7271	±0.0826 ^d^	18.5057	±0.0813 ^c^	28.9400	±0.0519 ^a^	26.3620	±0.1285 ^b^
	histidine	17.3273	±0.0918 ^b^	13.5823	±0.4245 ^c^	0.1331	±0.1484 ^d^	17.9267	±0.0526 ^c^
	isoleucine	58.6427	±0.0816 ^b^	46.9240	±0.0580 ^b^	8.5097	±0.0875 ^d^	20.4000	±0.0523 ^c^
**IR28**	leucine	38.6567	±0.0405 ^a^	31.2697	±0.0448 ^b^	0.1057	±0.0024 ^d^	38.8330	±0.0480 ^c^
	lysine	38.6567	±0.0405 ^b^	31.2697	±0.0448 ^b^	0.1057	±0.0024 ^d^	38.8330	±0.0480 ^c^
	methionine	4.8377	±0.0604 ^c^	7.5853	±0.0221 ^c^	0.3053	±0.0026 ^d^	7.1460	±0.0388 ^b^
	phenylalanine	35.6660	±0.0340 ^b^	30.5953	±0.0244 ^c^	0.9770	±0.0128 ^d^	37.3160	±0.0742 ^c^
	valine	25.3173	±0.0696 ^b^	20.1277	±0.2022 ^b^	1.3500	±0.3846 ^d^	25.6160	±0.0638 ^c^
	histidine	14.4810	±0.0216 ^c^	10.1870	±0.0992 ^d^	10.4380	±0.0736 ^c^	12.4347	±0.0691 ^d^
	isoleucine	48.5057	±0.0813 ^c^	34.4333	±0.0689 ^d^	35.0070	±0.0016 ^c^	43.2627	±0.1078 ^d^
**Pokkali**	leucine	31.7527	±0.0205 ^b^	23.6393	±0.0436 ^c^	23.3773	±0.0469 ^a^	28.4547	±0.0406 ^a^
	lysine	31.7527	±0.0205 ^c^	23.6393	±0.0436 ^d^	23.3773	±0.0466 ^c^	28.4547	±0.0406 ^d^
	methionine	2.6607	±0.0372 ^d^	1.8703	±0.0389 ^d^	2.7887	±0.1329 ^c^	3.6467	±0.0428 ^d^
	phenylalanine	30.1867	±0.0099 ^c^	24.1847	±0.0108 ^d^	22.9813	±0.0256 ^c^	29.4773	±0.0167 ^d^
	valine	20.9020	±0.1092 ^c^	15.0550	±0.1042 ^d^	15.0603	±0.0998 ^c^	17.9817	±0.0146 ^d^

**Table 2:** Effects of different salt stress conditions on non-essential amino acid profiles of four types of rice seedlings. The different letters of the means ± SDs, *n* = 3, indicate significant variations in the contents of the amino acids. However, Duncan’s multiple range test, *p* 0.05, shows that the means of the identical letters are not statistically different.

	Amino Acids Concentration (mg/g)								
	Amino Acids	Control		NaCl		CaCl_2_		MgCl_2_	
	alanine	3.8897	±0.09458 ^b^	5.8680	±0.1182 ^a^	6.3473	±0.0849 ^a^	8.8637	±0.1219 ^a^
	aspartic acid	0.0000	±0.0000 ^d^	0.0120	±0.0008 ^d^	0.0280	±0.0008 ^c^	12.6287	±0.0542 ^a^
	arginine	18.5940	±0.0077 ^d^	18.8767	±0.0205 ^d^	13.6407	±0.0073 ^b^	20.2863	±0.0383 ^b^
**Cheongcheong**	cystine	6.0297	±0.0499 ^b^	6.8760	±0.1232 ^a^	5.7897	±0.1353 ^c^	17.7533	±0.2000 ^a^
	glutamic acid	2.0557	±0.0328 ^b^	1.9530	±0.0751 ^b^	1.0663	±0.0407 ^c^	79.3577	±0.4542 ^a^
	proline	67.4487	±0.3886 ^b^	68.3933	±0.0062 ^a^	60.4013	±0.0849 ^b^	180.5450	±0.0666 ^a^
	serine	30.5667	±0.0262 ^a^	32.5203	±0.2946 ^a^	26.5350	±0.1382 ^b^	5.6793	±0.1636 ^c^
	tyrosine	32.7213	±0.0886 ^a^	30.8024	±0.0853 ^a^	31.3510	±0.0753 ^c^	41.6530	±0.0654 ^a^
	alanine	2.7193	±0.1678 ^c^	3.9213	±0.0575 ^b^	4.4190	±0.0686 ^b^	1.8937	±0.0869 ^d^
	aspartic acid	0.0943	±0.0030 ^c^	0.0297	±0.0012 ^c^	0.0400	±0.0008 ^b^	0.3170	±0.0008 ^b^
	arginine	26.8177	±0.1422 ^c^	31.1870	±0.0220 ^a^	22.9353	±0.0719 ^a^	16.9047	±0.0977 ^c^
**Nagdong**	cystine	3.4220	±0.1742 ^c^	4.6633	±0.1347 ^c^	8.0563	±0.1023 ^b^	7.9863	±0.0344 ^b^
	glutamic acid	0.8600	±0.0445 ^d^	0.8253	±0.0592 ^c^	1.9667	±0.0286 ^b^	50.7533	±0.2684 ^b^
	proline	37.1363	±0.0310 ^d^	42.9877	±0.0167 ^b^	67.5527	±0.1063 ^a^	0.4480	±0.0490 ^c^
	serine	17.7547	±0.0937 ^c^	17.7397	±0.2226 ^c^	28.4620	±0.0602 ^a^	46.1547	±0.2519 ^a^
	tyrosine	17.7763	±0.0459 ^d^	22.8867	±0.0294 ^b^	38.0600	±0.0432 ^a^	26.9487	±0.0365 ^b^
	alanine	6.3427	±0.3871 ^a^	1.5003	±0.3229 ^d^	0.0600	±0.0163 ^d^	0.7853	±0.1222 ^c^
	aspartic acid	7.5447	±0.0054 ^a^	0.3880	±0.0008 ^b^	0.0022	±0.0001 ^d^	0.2353	±0.0036 ^c^
	arginine	37.9410	±0.0820 ^a^	30.3133	±0.0981 ^b^	0.2370	±0.0453 ^c^	13.4747	±0.0850 ^d^
**IR28**	cystine	7.4310	±0.0488 ^a^	5.0977	±0.0036 ^b^	64.0577	±0.1019 ^a^	5.1280	±0.0523 ^c^
	glutamic acid	51.8383	±0.1194 ^a^	2.5927	±0.2237 ^a^	23.0097	±0.0725 ^a^	12.8890	±0.0954 ^c^
	proline	114.3420	±0.1197 ^a^	39.5077	±0.0825 ^c^	46.3053	±0.0849 ^c^	0.0073	±0.0012 ^d^
	serine	0.0480	±0.0021 ^d^	19.5293	±0.3328 ^b^	23.0097	±0.0725 ^c^	46.1023	±0.0761 ^a^
	tyrosine	22.4483	±0.0767 ^b^	20.9170	±0.0794 ^c^	36.3613	±0.0704 ^b^	25.0637	±0.0209 ^c^
	alanine	1.8300	±0.0973 ^d^	2.9263	±0.0547 ^c^	2.9950	±0.0063 ^c^	4.4933	±0.1069 ^b^
	aspartic acid	1.3100	±0.0488 ^b^	2.3160	±0.0024 ^a^	0.1417	±0.0012 ^a^	0.3000	±0.0008 ^b^
	arginine	31.7940	±0.1709 ^b^	22.3023	±0.0964 ^c^	22.9140	±0.1037 ^a^	27.8323	±0.0985 ^a^
**Pokkali**	cystine	1.4320	±0.0662 ^d^	1.9943	±0.0172 ^d^	2.3770	±0.0910 ^d^	1.7170	±0.0608 ^d^
	glutamic acid	1.5097	±0.0440 ^c^	0.7767	±0.1247 ^c^	0.4673	±0.0529 ^d^	0.7697	±0.0865 ^d^
	proline	41.5357	±0.0484 ^c^	31.6633	±0.0262 ^d^	31.3440	±0.0910 ^d^	38.3803	±0.0873 ^b^
	serine	21.3560	±0.0881 ^b^	13.8670	±0.1142 ^d^	14.5700	±0.0778 ^d^	17.0800	±0.0081 ^b^
	tyrosine	18.2497	±0.0406 ^c^	15.0227	±0.0165 ^d^	14.9750	±0.0094 ^d^	18.7513	±0.0433 ^d^

## Data Availability

The original contribution in the current work is included in the article, and any further questions should be referred to the corresponding author.
